# Bariatric Surgery and Lung Transplant Outcomes: Case Series and Insights from a Propensity-Matched Analysis at a High-Volume Transplant Center

**DOI:** 10.1007/s11695-025-07932-3

**Published:** 2025-05-28

**Authors:** Andrés Latorre-Rodriguez, Mark Shacker, Hesham Mohamed, Ross M. Bremner, Sumeet K. Mittal

**Affiliations:** 1 St. Joseph’s Hospital and Medical Center, Phoenix, AZ USA; 2 Escuela de Medicina y Ciencias de la Salud, Bogota D.C., Colombia; 3 Creighton University, Phoenix, AZ USA; 4 Mayo Clinic, Scottsdale, AZ USA

**Keywords:** Bariatric surgery, Metabolic surgery, Lung transplantation, Obesity, Chronic lung allograft dysfunction, Gastroesophageal reflux

## Abstract

**Background:**

An increasing number of patients with a history of bariatric surgery and advanced respiratory disease are presenting for lung transplantation (LTx). We aimed to describe and compare LTx outcomes between recipients with prior bariatric surgery and a matched control group at a high-volume lung transplant center.

**Methods:**

After IRB approval, we identified bilateral LTx recipients with a pre-LTx history of bariatric surgery (Roux-en-Y gastric bypass [RYGB], sleeve gastrectomy [SG], or laparoscopic adjustable gastric band [LAGB]). The institutional experience is reported as a case series. Furthermore, perioperative and mid-term transplant outcomes such primary graft dysfunction (PGD), antibody-mediated rejection (AMR), acute cellular rejection (ACR), chronic lung allograft dysfunction (CLAD)-free survival, and overall survival (OS) were compared to a 1-to-2 propensity score-matched control group.

**Results:**

Nine patients (median age: 65 years; 77.8% female) with a history of bariatric surgery (RYGB = 4, SG = 4, LAGB = 1) a median of 76 months before LTx were included. The median hospital length of stay (LOS) and ICU-LOS were similar to the control group (*n* = 18). Moreover, 1-, 2-, and 3-year OS in bariatric and control groups were similar (88.9%, 88.9%, and 66.7% vs. 100%, 86.7%, and 78%, respectively; *p* = 0.27). CLAD-free survival and rates of PGD, AMR, and ACR were also similar.

**Conclusions:**

Prior bariatric surgery may not affect overall or CLAD-free survival after bilateral LTx. Bariatric surgery for obesity treatment in patients with advanced lung diseases may improve their LTx candidacy without compromising early and mid-term transplant outcomes.

**Supplementary Information:**

The online version contains supplementary material available at 10.1007/s11695-025-07932-3.

## Introduction

Lung transplantation (LTx) is the only definitive treatment for end-stage lung disease [1]; however, it is complex and has a median survival of approximately 6 years, limited in most cases by chronic lung allograft rejection (CLAD) [2, 3]. Moreover, roughly 30% of patients will develop early complications such as primary graft dysfunction (PGD), antibody-mediated rejection (AMR), or acute cellular rejection (ACR) [4–6].

Due to the arduous postoperative course, careful candidate selection and candidate optimization have been proposed to ease morbidity and mortality [7, 8]. Obesity is a common medical condition among LTx candidates due to its increasing worldwide incidence, and severe obesity is usually a contraindication to LTx due to increased postoperative risks [9–12]. Therefore, an intervention that provides substantial weight reduction with a relatively uncomplicated postoperative recovery may improve LTx candidacy.

By the same token, with the growing popularity and use of surgical interventions for weight loss [13], the number of patients with prior bariatric surgery seeking LTx is expected to increase. Furthermore, bariatric surgery patients (particularly after sleeve gastrectomy [SG]) have a higher incidence of gastroesophageal reflux disease (GERD) and esophageal dysmotility, which are both associated with worse outcomes after LTx [14–16]. However, data on outcomes among LTx recipients with a history of bariatric surgery are limited. Therefore, we present a case series and compare early and mid-term LTx outcomes between recipients who had undergone pre-LTx bariatric surgery for weight loss and a matched control group.

## Methods

### Study Design and Setting

We present a case series of bilateral LTx recipients with a history of bariatric surgery before their LTx evaluation at a high-volume lung transplant center. In addition, we retrospectively compared the postoperative course and mid-term outcomes of these patients to a propensity score-matched control group. Patient demographics, history of bariatric surgery, listing conditions, and perioperative and postoperative course were retrospectively obtained through chart review or from prospectively maintained institutional databases. This research was approved by our institutional review board (PHXU-21–500-136–73-18; February 24, 2023) with waiver of written patient consent due to the study design. The STrengthening the Reporting of OBservational studies in Epidemiology (STROBE) statement and checklist were followed (**Supplementary material S1**).

### Sampling and Study Population

**Fig. 1:**
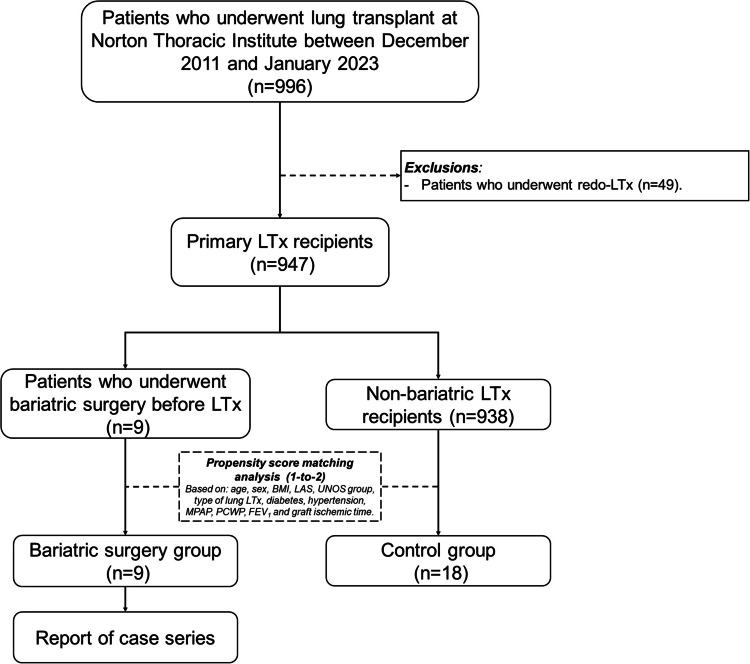
Study flow diagram. Abbreviations: *BMI *Body mass index, *FEV*_*1*_ forced expiratory volume in one second, *LAS* lung allocation score, *LTx* lung transplantation, *MPAP* Mean pulmonary artery pressure, *PCWP* Pulmonary capillary wedge pressure, *UNOS*: United Network for Organ Sharing

All adult patients (> 18 years) with history of bariatric surgery for weight loss (i.e., Roux-en-Y gastric bypass [RYGB], sleeve gastrectomy [SG], or laparoscopic adjustable gastric band [LAGB]) before LTx were identified using an institutional transplant listing repository; thus, sampling was completed by convenience. Patients were included in the study (i.e., bariatric surgery group) if they underwent bilateral LTx between December 2011 and January 2023 at our institution. Patients who underwent redo-LTx procedures were excluded. A 1-to-2 propensity score-matched control group was created from all transplanted patients during the same period considering 12 parameters: age, sex, body mass index (BMI), lung allocation score (LAS), United Network for Organ Sharing (UNOS) group, type of transplant, diabetes, hypertension, mean pulmonary artery pressure (MPAP), pulmonary capillary wedge pressure (PCWP), forced expiratory volume in one second (FEV1) at listing, and graft ischemic time. Figure 1 presents the study flow diagram.

### Outcomes and Definitions

Hospital length of stay (LOS), intensive care unit length of stay (ICU-LOS), incidence of PGD, AMR, and ACR within one year of LTx, CLAD-free survival, and overall survival (OS) were compared between the bariatric surgery and the matched control group. Standard definitions and diagnostic criteria were adopted from The International Society for Heart and Lung Transplantation guidelines [17–20].

### Patient Care, Testing, and Surveillance

#### Pulmonary Functional and Histological Testing

As standard practice, we obtain multiple pulmonary function tests (PFTs) before and after LTx using spirometry. After LTx, PFTs are conducted every 2–3 weeks during the first 6 months, then every month until year 2, and thereafter every 3–6 months. Moreover, all recipients undergo surveillance bronchoscopy (i.e., bronchoalveolar lavage and transbronchial biopsy) at 1, 3, 6, 9, and 12 months after LTx, and thereafter every 6 months. Additional tests are obtained as indicated.

#### Esophageal Functional Testing

Pre-LTx (i.e., during listing workup) and post-LTx (i.e., usually within 3–6 months after LTx) ambulatory esophageal 24-h pH monitoring, high-resolution manometry, and barium esophagram are completed following standard protocols and analyzed by an experienced foregut or thoracic surgeon.

#### Postoperative Patient Care

Most LTx recipients receive a similar immunosuppressive regimen for induction with IV methylprednisolone and basiliximab before perfusion of the lung allografts. A small subset of patients receives rituximab in combination with IV immunoglobulin. Immunosuppression in all cases is maintained with triple-drug therapy (steroids, mycophenolate mofetil, and tacrolimus).

### Sampling and Statistical Analysis

A maximum available sample size was achieved by convenience. Descriptive statistics were applied, reporting continuous variables as median (IQR) and categorical variables as count and proportion. Clinical characteristics and LTx outcomes of the bariatric patients were tabulated and presented as a case series. A propensity-matched control group was created using logistic regression and nearest neighbor without replacement. Balance was determined with an absolute standardized difference < 0.2. Differences in pre-specified outcomes were assessed using Fisher’s exact and Mann–Whitney *U* tests. Overall and CLAD-free survival were calculated via Kaplan–Meier curves and assessed with the log-rank test. Missing data were handled with pairwise deletion (available-case analysis), and *p*-values were adjusted by Bonferroni correction. Statistical significance was set a *p* < 0.05. All analyses were conducted using SPSS version 29.0 (IBM SPSS Statistics; Armonk, NY, USA) and R version 4.3.1 (R Foundation for Statistical Computing, Vienna, Austria).

## Results

### Demographic and Clinical Characteristics

A total of 996 LTx procedures were performed between December 2011 and January 2023; of these, 49 redo procedures were excluded. Nine patients with a history of bariatric surgery for weight loss before LTx were identified (7 [77.8%] women; median age at LTx, 65 [IQR 57–69] years). All bariatric procedures (RYGB, *n* = 4; SG, *n* = 4, LAGB, *n* = 1) were performed at other institutions. The median time from bariatric surgery to LTx was 76 (IQR 48–93) months. All but one patient was listed for LTx for restrictive lung disease (i.e., UNOS group D). Table 1 summarizes clinical characteristics and outcomes within the bariatric group (i.e., case series).

**Table 1:** Detailed clinical characteristics and outcomes of individuals in the bariatric group

**Pre-LTx characteristics**											**Post-LTx characteristics**													
**Age, Sex**	**Surgery Type**	**Surgery to LTx, mo**	**Diagnosis**	**UNOS Group**	**LAS**	**BMI kg/m**^**2**^	**CCI**	**HRM**	**DMS**	**HRM**		**DMS**	**ICU LOS, days**	**LOS, days**	**PGD**	**Worst PGD grade**	**AMR**	**ACR**	**Worst ACR grade**	**CLAD**	**FU time, mo**	**BMI at last FU kg/m**^**2**^	**Status**	**Cause of death**
66, F	RYGB	169	UIP/IFP	D	40.62	27.4	5	Inconclusive IEM	3.1	Normal		2.3	12	22	No	G0	No	Yes	A1	No	31	21.9	Deceased	Sepsis
40, F	RYGB	76	COVID-Fibrosis	D	62.19	27.0	1	-	-	Normal		0.7	7	32	Yes	G3	No	No	A0	No	10	30.1	Alive	-
77, F	RYGB	63	ILD/HP	D	43.45	30.9	5	Inconclusive IEM	25.9	Normal		1.2	17	50	Yes	G3	No	No	A0	No	45	23.3	Deceased	Unknown
69, F	RYGB	151	COPD	A	37.10	19.9	4	DES	10.7	-		-	13	40	Yes	G1	No	No	A0	No	3	18.8	Deceased	Type A aortic aneurysm
57, F	SG	93	UIP/IFP	D	37.80	28.9	2	Inconclusive EGJOO	23.2	Inconclusive EGJOO			11	41	No	G0	No	No	A0	No	135	15.5	Alive	-
64, F	SG	48	NSIP/UIP	D	42.42	30.1	4	Normal	24.5	Normal		25.3	9	22	Yes	G2	No	Yes	A2	No	3	26.2	Alive	-
55, F	SG	43	NSIP/UIP	D	37.80	32.5	2	Inconclusive IEM	15.9	Inconclusive IEM		30.9	7	31	Yes	G3	Yes	Yes	A2	Yes	18	39.1	Alive	-
73, M	LB	12	NSIP/UIP	D	46.50	22.7	4	Inconclusive EGJOO	0.6	-		-	8	40	Yes	G2	No	Yes	A1	No	20	29.1	Alive	-
65, M	SG	76	UIP/IFP	D	85.03	26.7	4	-	-	Inconclusive IEM		88.6	15	35	Yes	G3	No	No	A0	Yes	59	28.3	Deceased	Respiratory failure

Abbreviations: *ACR*, acute cellular rejection; *AMR*, antibody mediated rejection; *BMI*, body mass index; *CCI*, Charlson comorbidity index; *CLAD*, chronic lung allograft dysfunction; *COPD*, chronic obstructive pulmonary disease; *DES*, distal esophageal spasm; *DMS*, DeMeester score; *EGJOO*, esophagogastric outflow obstruction; *F*, female; *FU*, follow-up; *HRM*, high-resolution manometry; *HP*, hypersensitivity pneumonitis; *ICU*, intensive care unit; *IEM*, ineffective esophageal motility; *IFP*, idiopathic pulmonary fibrosis; *ILD*, interstitial lung disease; *LAS*, lung allocation score; *LB*, LapBand; *LOS*, length of stay; *M*, male; *NSIP*, nonspecific interstitial pneumonia; *LTx*, lung transplantation; *PGD*, primary graft dysfunction; *RYGB*, Roux-en-Y gastric bypass; *SG*, sleeve gastrectomy; *UIP*, usual interstitial pneumonia; *UNOS*, United Network for Organ Sharing

The control group was developed from the remaining 938 primary LTx recipients during the same period. After propensity-matching, 18 patients formed this group. The median age at LTx was 67.5 (IQR 59.5–70.8) years, 17 (94.4%) were female, and most had a restrictive lung disease (88.9%). The age, pre-LTx BMI, history of diabetes and hypertension, UNOS group, LAS, and type of LTx were adequately balanced and remarkably similar between groups. Similarly, although not reaching Cohen’s d cutoff for a small effect size, the remaining baseline characteristics (sex, history of smoking, MPAP, PCWP, FEV1, and graft ischemia time) were also comparable (Supplementary material S2).

### Pulmonary and Esophageal Function

The DeMeester score (DMS), total acid exposure time (AET), and the proportion of patients with impaired esophageal body peristalsis were similar between the groups before and after LTx (Supplementary material S3). However, within the bariatric surgery group after LTx, patients in the SG subgroup had a higher median DMS and AET than those in the RYGB subgroup (30.9 vs. 1.2 and 9.8% vs. 0.1%, respectively), suggesting that the SG subgroup was more prone to pathological reflux and its related complications (Supplementary material S4).

Bariatric patients and controls demonstrated similar pulmonary function before LTx, and most patients improved after LTx. Regardless of the group, pulmonary function was stable among patients who reached 1-, 2-, and 3-year post-LTx follow-ups; nevertheless, a slight trend toward functional decline was noted thereafter (i.e., 5-year follow-up) among the survivors (Supplementary material S4).

### Hospital and ICU Length of Stay

Both the hospital-LOS and ICU-LOS after LTx were slightly longer in the bariatric surgery group than the control group, but this difference was not statistically significant (median LOS: 35 (IQR 31–40) vs. 24 (IQR 17–30) days; median ICU-LOS: 11 (IQR 8–13) vs. 9 (IQR 5–11) days (Table 2)). Moreover, the RYGB and SG subgroups had a median LOS of 36 (IQR 27–45) and 33 (IQR 26.5–38) days, respectively, and a median ICU-LOS of 12.5 (IQR 9.5–15) and 10 (IQR 8–13) days, respectively (Supplementary material S4).

**Table 2:** Perioperative and lung transplant outcomes among lung transplant recipients with history of bariatric surgery and a control group

	**Bariatric surgery group (*n* = 9)**	**Control group (*n* = 18)**	**p-value**
**Perioperative outcomes**			
Length of ICU stay, days	11 [8, 13]	9 [5, 11]	0.11
Hospital length of stay, days	35 [31, 40]	24 [17, 30]	0.19
**Post-LTx outcomes**			
Any grade of PGD within 72 h after LTx	7 (77.8)	10 (55.6)	0.42
PGD grade 3 within 72 h after LTx	4 (44.4)	6 (33.3)	0.68
AMR within 1 year after LTx	1 (11.1)	4 (22.2)	0.57
Any grade of ACR within 1 year after LTx	4 (44.4)	3 (16.7)	0.12
Highest grade of ACR documented			
*No evidence of ACR (grade A0)*	5 (55.6)	16 (88.8)	0.22
*Mild ACR (grade A1)*	2 (22.2)	1 (5.6)	
*Moderate ACR (grade A2)*	2 (22.2)	1 (5.6)	
*Severe ACR (grade A3)*	0 (0)	0 (0)	
Evidence of CLAD at any time after LTx	2 (22.2)	3 (16.7)	0.73

All values are presented as counts and proportions or medians and interquartile ranges

Abbreviations: *ACR*, acute cellular rejection; *AMR*, antibody mediated rejection; *CLAD*, chronic lung allograft dysfunction; *ICU*, intensive care unit; *LOS*, length of stay; *LTx*, lung transplantation; *PGD*, primary graft dysfunction

### Incidence of PGD, AMR, and ACR

There was a slightly higher (not statistically significant) incidence of any grade of PGD within the first 72 h after LTx among the bariatric group compared to controls (77.8% vs. 55.6%, *p* = 0.42) (Table 2). The incidence of severe PGD (i.e., grade 3) and AMR were similar between the groups (PGD: 44.4% vs. 33.3%, *p* = 0.68; AMR: 11.1% vs. 22.2%, *p* = 0.57). The incidence of ACR among the bariatric group and controls was 44.4% and 16.7%, respectively (*p* = 0.12), but all cases of ACR were minimal (A1) or mild (A2) (Tables 1 and 2).

### Survival Analysis

**Fig. 2:**
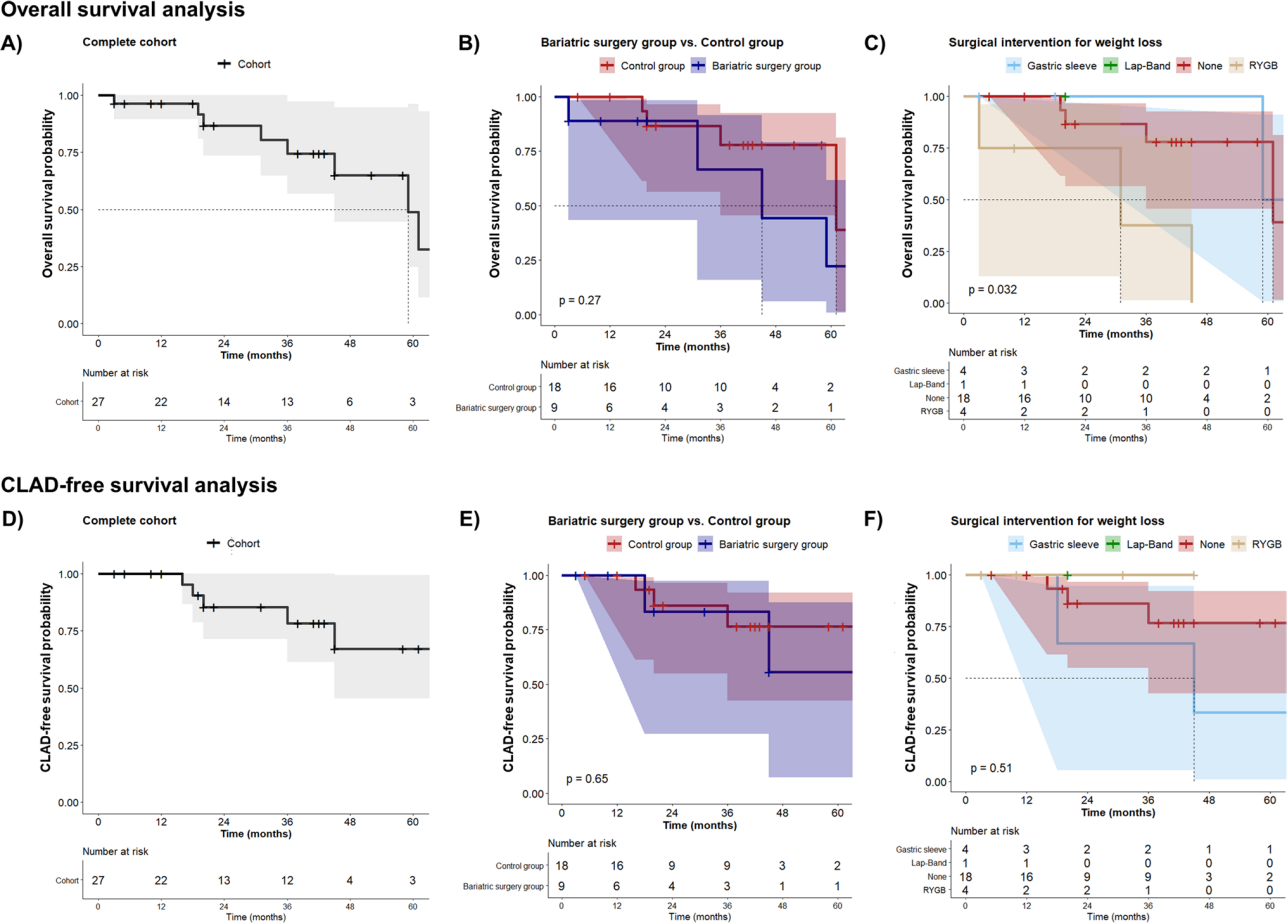
Kaplan–Meier curves. **A** Overall survival for the entire cohort, **B** stratified overall survival for bariatric surgery and control groups, **C** stratified overall survival according to the type of bariatric intervention, **D** CLAD-free survival for the entire cohort, **E** stratified CLAD-free survival for bariatric surgery and control groups, **F** stratified CLAD-free survival according to the type of bariatric intervention. Of note, subgroup survival analyses and plots (i.e., between type of interventions) are recommended to be taken as descriptive observations rather than inferential analyses. Abbreviations: CLAD: chronic lung allograft dysfunction; RYGB: Roux-en-Y gastric bypass

Four (44.4%) patients with history of bariatric surgery and 4 (22.2%) in the control group died during the observation period. The median overall survival of the entire cohort was 59 months (Fig. 2A), whereas the median survival for the bariatric surgery and control groups was 45 months and 61 months, respectively. The cumulative survival in the bariatric group at 1, 2, and 3 years was 88.9%, 88.9%, and 66.7% compared to 100%, 86.7%, and 78% in the control group (log-rank test, *p* = 0.27, Fig. 2B). The causes of death within the RYGB subgroup (*n* = 3) were immunosuppression-related sepsis (*n* = 1), acute type A aortic aneurysm dissection (*n* = 1), and unknown (*n* = 1), and the cause of death in the SG patient was CLAD. Deaths in the control group were COVID-19 sequelae (*n* = 2) and CLAD-related respiratory failure (*n* = 2). Notably, patients in the RYGB subgroup had lower overall survival than those in the SG subgroup (Fig. 2C). Five (18.5%) patients were diagnosed with CLAD during the study period (Fig. 2D). CLAD-free survival appeared to be similar between the bariatric surgery group and controls (Fig. 2E) but slightly worse among the SG subgroup (Fig. 2F).

## Discussion

Bariatric surgery for the treatment of severe obesity is rapidly increasing; Approximately 280,000 procedures were performed in the United States in 2022, a 6.5% increase from 2021 [13]. Likewise, lung transplants rose by 6.8% over the same period, from 2,569 to 2,743 [21]. Thus, it is becoming more common for lung transplant centers to evaluate patients with a history of bariatric surgery. This intersection requires particular attention, as bariatric surgery may contribute to the development of GERD and/or esophageal dysmotility—both recognized risk factors for ACR and CLAD.

Although the effects of bariatric surgery on LTx outcomes are largely unknown, there are multiple mechanisms by which bariatric surgery may improve patient candidacy for LTx and lead to outcomes similar to those of non-obese LTx recipients. Bariatric surgery may facilitate the resolution of obesity-associated structural changes in the thoracoabdominal region and decrease the systemic pro-inflammatory signaling status [22]. After bariatric surgery, an overall increase in pulmonary functional capacity (especially in patients with interstitial lung disease) is expected due to an improvement in diaphragmatic and thoracic cage mobility [22–24]. Such changes may also delay the need for lung transplant listing. Furthermore, weight loss can reduce the overproduction of obesity-related cytokines that can induce a continuous pro-inflammatory state and predispose patients to develop bronchial hyperresponsiveness [25].

By 2020, only three centers had reported their experience [22, 26, 27], including 28 patients with end-stage respiratory disorders (mostly interstitial lung disease [*n* = 26, 92.8%]) who were not considered candidates for LTx due to obesity [24]. Of these patients, after bariatric surgery (RYGB = 18, SG = 9, LAGB = 1), eight (28.6%) were listed for LTx and two (7%) underwent LTx. One of these recipients had reached a survival of 88 months without complications [22]. Moreover, four (14.3%) patients improved clinically, precluding the need for listing. These data support the safety of bariatric surgery even in patients with severe associated medical problems as well as the important role of surgical weight loss management to achieve LTx listing and eventually undergo LTx; however, they do not directly address LTx outcomes in patients with a history of bariatric surgery.

Our experience including nine LTx recipients with a history of pre-LTx bariatric surgery may provide significant and novel insights. Overall, compared to a matched control group, these patients did not perform significantly worse after LTx. This suggests that a history of bariatric surgery should not be a contraindication to LTx. Nevertheless, there is a need for further clarification of potential post-LTx risks derived from prior bariatric procedures. Despite the limited small sample size, we observed that patients in the RYGB subgroup had worse overall survival than those in the SG subgroup, but paradoxically a trend toward increased rates of ACR, CLAD, and worse CLAD-free survival was seen among the SG subgroup.

The shorter median overall survival in the RYGB subgroup may be attributed to early, non-LTx-related deaths (e.g., type A aneurysm dissection) or deaths unrelated to graft dysfunction (e.g., sepsis-induced multiorgan failure). Additionally, anatomical changes from RYGB could impact medication pharmacokinetics and absorption (including that of immunosuppression therapy), which warrants tailored strategies—such as serum monitoring of medication levels—to ensure therapeutic efficacy and reduce risks of toxicity or undertreatment [28]. Meanwhile, in patients with a history of SG, the higher risk of GERD-related aspiration and subsequent allograft injury may explain the trend toward an increased CLAD incidence and worse CLAD-free survival in this group. For these patients, regular functional esophageal and respiratory surveillance may be advised to detect complications early. Furthermore, early surgical management of GERD after LTx (i.e., before 180 days) has been proven to be safe and is a critical step to improve patient outcomes [29]. Another important aspect of bariatric surgery to consider is the narrow and altered anatomy of the SG, which makes postoperative nutrition management more difficult (i.e., J or G tube placement) than that after RYGB. Thus, the potential complexity of postoperative enteral access for SG patients theoretically places them in a different risk category. Finally, patients who have undergone RYGB may have an increased risk of developing a marginal ulcer due to chronic steroid use after LTx [30].

Our study has several limitations including a single-center design with a small, heterogeneous sample (i.e., multiple bariatric surgery techniques), limiting further statistical analyses (e.g., competing events in survival analysis) and generalizability. Given a small sample size and reduced statistical power (~ 20.5% based on a post hoc calculation for overall survival), the *p*-values were reported only for pre-specified outcomes to minimize spurious findings. However, this is the largest report to date, and we propose using these results to guide future larger studies. Furthermore, as the bariatric procedures were performed at different institutions, we were not able to retrieve specific anthropometric or operative data. Moreover, the median time from bariatric surgery to lung transplantation varied among included patients, which may have influenced both weight loss maintenance and graft outcomes. Lastly, the LTx procedures were performed in a wide observation period (i.e., 2011–2023), which makes the postoperative course of each patient unique and susceptible to variations in standard practices.

## Conclusion

The postoperative course—measured by hospital-LOS and ICU-LOS as well as the incidence of PGD, AMR, ACR, and CLAD—appear to be similar between LTx recipients with a history of bariatric surgery for weight loss and matched controls. Therefore, a history of bariatric surgery should not preclude LTx. However, some trends indicating variations in post-LTx outcomes based on the type of bariatric intervention were observed. Thus, we recommend a multidisciplinary approach both pre- and post-LTx for these patients, involving specialists in bariatric surgery, thoracic surgery, pulmonology, nutrition, and pharmacy to address specific concerns that may arise (i.e., weight control, nutrition and medication absorption, GERD and aspiration, and enteral complications such as delayed gastric emptying). Further research involving larger sample sizes is needed.

## Supplementary Information

Below is the link to the electronic supplementary material.

Supplementary file1 (PDF 455 KB)

## Data Availability

The data used for this study is stored in a secure REDcap® dataset that cannot be shared outside of those authorized as research staff per protocol. Access to this dataset requires IRB approval; if needed, direct to the corresponding author.
