# Correction: Bariatric Surgery and Lung Transplant Outcomes: Case Series and Insights from a Propensity-Matched Analysis at a High-Volume Transplant Center

**DOI:** 10.1007/s11695-025-07979-2

**Published:** 2025-06-13

**Authors:** Andrés R. Latorre-Rodriguez, Mark Shacker, Hesham Mohamed, Ross M. Bremner, Sumeet K. Mittal

**Affiliations:** 1 St. Joseph’s Hospital and Medical Center, Phoenix, AZ USA; 2 Universidad del Rosario, Bogotá D.C., Colombia; 3 Creighton University, Phoenix, AZ USA; 4 Mayo Clinic, Scottsdale, AZ USA

**Correction: Obesity Surgery**

10.1007/s11695-025-07932-3

The name and affiliation of the first author was published incorrectly. “Andrés Latorre-Rodriguez” should be corrected to “Andrés R. Latorre-Rodriguez”, and the second affiliation “Escuela de Medicina y Ciencias de la Salud, Bogota D.C. Colombia” should be corrected to “Universidad del Rosario, Bogotá D.C., Colombia.”

