# Supplementary material for: Bariatric Surgery and Lung Transplant Outcomes: Case Series and Insights from a Propensity-Matched Analysis at a High-Volume Transplant Center
Source: Obes Surg. 2025 May 28;35(7):2436–44. doi: 10.1007/s11695-025-07932-3 (PMC12271277; doi:10.1007/s11695-025-07932-3)
Supplement: Supplementary file 1 — Supplementary file1 (PDF 455 KB) [file 11695_2025_7932_MOESM1_ESM.pdf]

Supplementary material S1. The Strengthening the Reporting of Observational Studies in Epidemiology (STROBE) Statement: guidelines for reporting observational studies.

	Item No	Recommendation	Page No
**Title and abstract**	1	(*a*) Indicate the study's design with a commonly used term in the title or the abstract	Pg. 1
		(*b*) Provide in the abstract an informative and balanced summary of what was done and what was found	Pg. 3
**Introduction**			
Background/rationale	2	Explain the scientific background and rationale for the investigation being reported	Pg. 5
Objectives	3	State specific objectives, including any prespecified hypotheses	Pg. 5-6
**Methods**			
Study design	4	Present key elements of study design early in the paper	Pg. 6
Setting	5	Describe the setting, locations, and relevant dates, including periods of recruitment, exposure, follow-up, and data collection	Pg. 6
Participants	6	(*a*) *Cohort study*—Give the eligibility criteria, and the sources and methods of selection of participants. Describe methods of follow-up<br>*Case-control study*—Give the eligibility criteria, and the sources and methods of case ascertainment and control selection. Give the rationale for the choice of cases and controls<br>*Cross-sectional study*—Give the eligibility criteria, and the sources and methods of selection of participants	Pg. 6-7
		(*b*) *Cohort study*—For matched studies, give matching criteria and number of exposed and unexposed<br>*Case-control study*—For matched studies, give matching criteria and the number of controls per case	Pg. 7
Variables	7	Clearly define all outcomes, exposures, predictors, potential confounders, and effect modifiers. Give diagnostic criteria, if applicable	Pg. 7
Data sources/ measurement	8*	For each variable of interest, give sources of data and details of methods of assessment (measurement). Describe comparability of assessment methods if there is more than one group	Pg. 5-7
Bias	9	Describe any efforts to address potential sources of bias	Pg. 8
Study size	10	Explain how the study size was arrived at	

Quantitative variables	11	Explain how quantitative variables were handled in the analyses. If applicable, describe which groupings were chosen and why	Pg. 6-7
Statistical methods	12	(*a*) Describe all statistical methods, including those used to control for confounding	Pg. 8-9
		(*b*) Describe any methods used to examine subgroups and interactions	Pg. 8-9
		(*c*) Explain how missing data were addressed	Pg. 8-9
		(*d*) *Cohort study*—If applicable, explain how loss to follow-up was addressed<br>*Case-control study*—If applicable, explain how matching of cases and controls was addressed<br>*Cross-sectional study*—If applicable, describe analytical methods taking account of sampling strategy	N/A
		(*e*) Describe any sensitivity analyses	Pg. 8-9

Results

Participants	13*	(a) Report numbers of individuals at each stage of study—eg numbers potentially eligible, examined for eligibility, confirmed eligible, included in the study, completing follow-up, and analysed	Pg. 9 and Fig 1.
		(b) Give reasons for non-participation at each stage	
		(c) Consider use of a flow diagram	
Descriptive data	14*	(a) Give characteristics of study participants (eg demographic, clinical, social) and information on exposures and potential confounders	Pg. 9, Table 1.
		(b) Indicate number of participants with missing data for each variable of interest	Described in Tables 1-2 + Sup material.
		(c) *Cohort study*—Summarise follow-up time (eg, average and total amount)	Pg. 9
Outcome data	15*	*Cohort study*—Report numbers of outcome events or summary measures over time	Pg. 9-12
		Case-control study—Report numbers in each exposure category, or summary measures of exposure	N/A
		Cross-sectional study—Report numbers of outcome events or summary measures	N/A
Main results	16	(*a*) Give unadjusted estimates and, if applicable, confounder-adjusted estimates and their precision (eg, 95% confidence interval). Make clear which confounders were adjusted for and why they were included	Pg. 9-12 (Unadj est.) – N/A confounder est.

		(*b*) Report category boundaries when continuous variables were categorized	Pg. 9-12 and Table 2. Suppl material.
		(*c*) If relevant, consider translating estimates of relative risk into absolute risk for a meaningful time period	N/A
Other analyses	17	Report other analyses done—eg analyses of subgroups and interactions, and sensitivity analyses	Pg. 9-12. Supplementary material S2-4.
**Discussion**			
Key results	18	Summarise key results with reference to study objectives	Pg. 12-13
Limitations	19	Discuss limitations of the study, taking into account sources of potential bias or imprecision. Discuss both direction and magnitude of any potential bias	Pg. 14-15
Interpretation	20	Give a cautious overall interpretation of results considering objectives, limitations, multiplicity of analyses, results from similar studies, and other relevant evidence	Pg. 12-15
Generalizability	21	Discuss the generalizability (external validity) of the study results	Pg. 14
**Other information**			
Funding	22	Give the source of funding and the role of the funders for the present study and, if applicable, for the original study on which the present article is based	Pg. 16

*Give information separately for cases and controls in case-control studies and, if applicable, for exposed and unexposed groups in cohort and cross-sectional studies.

Supplementary material S2. Demographic and clinical characteristics at lung transplant listing of the bariatric surgery and control groups.

	**Bariatric surgery group (n=9)**	**Control group (n=18)**	**S.M.D**
**Demographics**			
Age, years	65 [57, 69]	67.5 [59.5, 70.8]	0.16
Sex, (M:F)	1:17	2:7	0.25
BMI, kg/m^2	27.4 [26.7, 30.1]	28.6 [26.2, 30.1]	0.05
**Clinical characteristics**			
Smoking	6 (66.7)	10 (55.6)	†
Diabetes	2 (22.2)	3 (16.7)	0.14
Hypertension	5 (55.6)	9 (50)	0.11
UNOS group:			
A, Obstructive disorders	1 (11.1)	1 (5.6)	0.06
B, Pulmonary hypertension	0 (0)	1 (5.6)	
C, Cystic fibrosis	0 (0)	0 (0)	
D, Restrictive disorders	8 (88.9)	16 (88.9)	
Lung allocation score	42.4 [37.8, 46.5]	46.4 [37.7, 52.9]	0.09
MPAP, mmHg	24 [20, 27]	21.5 [18.3, 23.8]	0.60
PCWP, mmHg	12 [7, 12]	8 [5, 11]	0.45
FEV_1, L	1.38 [1.13, 1.79]	1.33 [0.71,1.51]	0.45
Cardiac output, L/min	6.15 [5.40, 6.37]	5.56 [4.69, 6.02]	†
Cardiac index, L/min/m^2	3.23 [3.12, 3.50]	3.01 [2.72, 3.40]	†
**Transplantation characteristics**			
Bilateral LTx	9 (100)	18 (100)	0.00
Graft ischemic time, min	260 [187, 287]	216 [151, 298]	0.23

All values are presented as counts and proportions or medians and interquartile ranges, unless otherwise specified.

Note: Due to the small sample size, the likelihood of large effect sizes and Type I error increases. Therefore, p-values are reported only for pre-specified outcomes of interest.

Abbreviations: BMI: body mass index; **FEV_1:** forced expiratory volume in the first second; **LTx:** lung transplantation; **MPAP:** mean pulmonary arterial pressure; **PCWP:** pulmonary capillary wedge pressure; **S.M.D:** standardized mean difference.

Supplementary material S3. Esophageal and pulmonary functional testing before and after lung transplant.

	Bariatric surgery group (n=9)	Control group (n=18)
**Pre-LTx esophageal functional tests**		
DeMeester score	15.9 [3.1, 24.5]	11.6 [0.9, 73.0]
Total AET, %	3.7 [0.6, 5.8]	3.1 [0.2, 12.3]
Impaired motility †	2/7 (28.6)	6/14 (42.8)
**Post-LTx esophageal functional tests**		
DeMeester score	13.8 [1.2, 30.9]	7.4 [3.9, 32.3]
Total AET, %	3.9 [0.1, 9.8]	2.4 [0.7, 11.2]
Impaired motility †	2/7 (28.6)	3/16 (18.8)
**Pre-LTx pulmonary functional tests**		
FEV_1, L	1.38 [1.13, 1.79]	1.33 [0.71, 1.51]
FEV_1%	54 [47, 74]	62.5 [37, 72]
**Post-LTx pulmonary functional tests**		
1-year FEV_1, L	1.99 [1.88, 2.15]	2.23 [1.73, 2.57]
1-year FEV_1%	81.3 [69, 93]	96 [82.5, 108.5]
2-year FEV_1, L	2.05 [1.89, 2.39]	2.15 [1.73, 2.53]
2-year FEV_1%	89.5 [78.9, 91]	96.5 [82.5, 108.5]
3-year FEV_1, L	2.09 [1.89, 2.39]	2.08 [1.50, 2.24]
3-year FEV_1%	67 [57, 77]	83.5 [76, 101]
5-year FEV_1, L	1.80 [1.54, 2.05]	1.51 [1.1, 1.89]
5-year FEV_1%	64 [49, 79]	75 [54, 110]

All values are presented as counts and proportions or medians and interquartile ranges.

*Data available for 21 of 27 patients.

**Data available for 23 of 27 patients. §Representative data from 23, 18, 12, and 5 patients at 1-, 2-, 3- and 5-year follow-ups, respectively.

†Diagnosis of conclusive/inconclusive ineffective esophageal motility or absent contractility according to criteria established by the Chicago Classification v4.0.

Note: Denominators were adjusted to cases with complete information as appropriate. Due to the small sample size, the likelihood of large effect sizes and Type I error increases. Therefore, p-values are reported only for pre-specified outcomes of interest.

Abbreviations: AET: acid exposure time; **FEV_1:** forced expiratory volume in the first second; **FEV_1%:** predicted forced expiratory volume in the first second; **LTx:** lung transplantation.

Supplementary material S4. Subgroup comparison. Post-LTx functional esophageal and pulmonary tests as well as ICU and hospital LOS, incidence of PGD, AMR, ACR and CLAD according to the type of bariatric intervention.

	**RYGB (n=4)**	**SG (n=4)**
**Esophageal functional testing**		
DeMeester score	1.2 [0.7, 2.3]	30.9 [25.3, 88.6]
Total AET, %	0.1 [0.1, 0.5]	9.8 [7.4, 27.8]
Impaired motility †	0/3 (0)	2/4 (50)
**Pulmonary functional testing**		
1-year FEV_1, L	1.99 [1.98, 2.07]	2.23 [1.73, 2.57]
1-year FEV_1%	93 [81.3, 98]	96 [82.5, 108.5]
2-year FEV_1, L	1.96 [1.89, 2.04]	2.15 [1.73, 2.53]
2-year FEV_1%	89.5 [89, 90]	96.5 [82.5, 108.5]
3-year FEV_1, L	-	2.08 [1.50, 2.24]
3-year FEV_1%	-	83.5 [76, 101]
5-year FEV_1, L	-	1.51 [1.1, 1.89]
5-year FEV_1%	-	75 [54, 110]
**Perioperative outcomes**		
Length of ICU stay, days	12.5 [9.5, 15]	10 [8, 13]
Hospital length of stay, days	36 [27, 45]	33 [26.5, 38]
**Post-LTx outcomes**		
Any grade of PGD within 72 hrs after LTx	3 (75)	3 (75)
AMR within 1 year after LTx	0 (0)	1 (25)
Any grade of ACR within 1 year after LTx	1 (25)	2 (50)
Evidence of CLAD at any time after LTx	0 (0)	2 (50)

All values are presented as counts and proportions or medians and interquartile ranges.
Note: Due to the small sample size, the likelihood of large effect sizes and Type I error increases. Therefore, p-values are reported only for pre-specified outcomes of interest.
Abbreviations: ACR: acute cellular rejection; **AET:** acid exposure time; **AMR:** antibody mediated rejection; **CLAD:** chronic lung allograft dysfunction; **FEV1:** forced expiratory volume in the first second; **FEV1%:** predicted forced expiratory volume in the first second; **ICU:** intensive care unit; **LOS:** length of stay; **LTx:** lung transplantation; **PGD:** primary graft dysfunction.
